# Isolation and Identification of Antifungal Polyketones from *Bacillus velezensis* DJ1 and Their Biocontrol Potential Against Corn Stalk Rot

**DOI:** 10.3390/biology14101436

**Published:** 2025-10-17

**Authors:** Meng Sun, Wanjia Du, Jialing Zhang, Yan Xu, Zixuan Wang, Lu Zhou, Gengxuan Yan, Shumei Zhang

**Affiliations:** Institute of Microbiology, Heilongjiang Academy of Sciences, Harbin, 150010, China; 15945345926@163.com (M.S.);

**Keywords:** *Fusarium graminearum*, microbial biocontrol, plant fungal disease, secondary metabolites

## Abstract

**Simple Summary:**

Corn, a major global food crop, is often damaged by *Fusarium graminearum*, which causes corn stalk rot and leads to serious yield losses. The objective of this study was to clarify the antifungal activity and underlying antifungal mechanism of polyketone compounds produced. In this study, the antifungal activity of DJ1-derived polyketones against *F. graminearum* were evaluated, while their effects on the morphological and functional characteristics of the fungus were examined. Additionally, the efficacy of these polyketones in reducing corn stalk rot incidence was assessed in a greenhouse, and their impacts on the soil microbial community were investigated. Results showed that DJ1 polyketones exerted effective inhibitory effects on *F. graminearum*, induced morphological and plasma membrane damage, and significantly reduced the occurrence of corn stalk rot with a disease control efficacy of 44.33%. Meanwhile, the polyketones did not disrupt the overall balance of the soil microbial community. Structural identification revealed that the bioactive components with antifungal activity were identified as difficidin and bacillaene. This research provides an effective strategy for the biocontrol of corn stalk rot.

**Abstract:**

Corn stalk rot, caused by *Fusarium graminearum*, severely threatens corn production, while chemical fungicides lead to resistance and environmental risks. Thus, exploring environmentally friendly biocontrol agents is crucial. Though *Bacillus* polyketones have antifungal activity, their active components, corn stalk rot biocontrol potential and rhizosphere impacts remain unclear. This study isolated polyketones from *Bacillus velezensis* DJ1, assessed their antifungal activity and mechanism against *F. graminearum*, verified greenhouse efficacy, analyzed rhizosphere microbiota, and identified active components via HPLC-HDMS Q-TOF. The results showed that DJ1 polyketones showed dose-dependent inhibition by disrupting *F. graminearum’s* mycelium and membrane, with 44.33% greenhouse control efficacy. They did not disturb rhizosphere balance, only increasing Bacteroidota and *DYGX01,* and the active components were difficidin and bacillaene. These findings confirm DJ1 polyketones as promising eco-friendly biocontrol agents, providing a new strategy for sustainable corn stalk rot management.

## 1. Introduction

Corn (*Zea mays* L.), as a globally crucial food and cash crop, faces persistent threats from various diseases that compromise its safe production [1,2]. Among these, corn stalk rot has emerged as one of the most devastating agricultural diseases limiting corn yield and quality, primarily due to its cryptic onset and rapid dissemination [3]. This disease is predominantly caused by *Fusarium graminearum*, a soil-borne and seed-transmitted pathogen that invades the basal stalks of corn plants [4,5]. Following infection, the pathogen induces stalk rot and subsequent lodging, resulting in substantial yield losses.

Chemical fungicides have been the primary strategy for managing corn stalk rot. However, the extensive and long-term application of these chemicals not only accelerates the development of fungicide resistance but also leads to soil contamination and adverse ecological impacts [3,5]. Consequently, the development of environmentally benign, target-specific biocontrol agents (BCAs) with low risk of resistance evolution have become a core focus in current research on corn stalk rot control.

While numerous microorganisms have been investigated for their biocontrol potential against plant pathogens [6], members of the *Bacillus* spp. have received considerable attention due to their exceptional stress tolerance, broad host range, and favorable biosafety profiles [7,8,9]. Notably, *Bacillus* spp. are capable of producing a diverse array of bioactive metabolites with potent antibacterial and antifungal activities [10,11,12,13,14,15,16]. Among these, polyketones represent a large and structurally diverse class of natural products biosynthesized via the PKS pathway [17], including bacillaene [18], difficidin [19], and macrolactin [14]. These compounds exhibit versatile biological activities, with well-documented roles in both inducing plant resistance to pathogens and directly inhibiting plant diseases. They exhibit unique structural diversity, enabling targeted interactions with pathogenic fungi. In addition, polyketones offer potential as novel fungicides, addressing the critical issue of pathogen resistance to conventional chemicals. These characteristics make polyketones a more promising candidate for corn stalk rot biocontrol. Notably, accumulating evidence has demonstrated the potent inhibitory effects of these *Bacillus*-derived polyketones against a broad spectrum of plant pathogenic microorganisms [20,21,22]. Nevertheless, research documenting the antifungal effects of *Bacillus velezensis*-produced polyketones on *F. graminearum* remains remarkably limited.

The *B. velezensis* strain DJ1 focused on in this study was isolated from rhizosphere soil in a previous survey. Notably, DJ1 was initially screened for its strong inhibitory activity against *F. graminearum*, outperforming 12 other tested strains. Furthermore, whole-genome sequencing of DJ1 revealed a complete PKS gene cluster, confirming its ability to synthesize polyketones. Additionally, as a rhizosphere-isolated strain, DJ1 exhibits strong adaptability to soil environments, ensuring its polyketones maintain activity in practical applications. These traits collectively make DJ1 an ideal strain for exploring polyketone-based biocontrol of corn stalk rot.

Based on the above background, the central hypothesis of this study is that the polyketone metabolites produced by *B. velezensis* DJ1 are the primary contributors to its observed antifungal activity against *F. graminearum*. To test this hypothesis, we employed an activity-guided fractionation strategy to isolate and purify the bioactive polyketone components from the fermentation broth of DJ1. The structures of the purified compounds were then elucidated. Furthermore, we systematically evaluated their antifungal efficacy both in vitro and in vivo. The potential mode of action was also investigated by examining the effects of these polyketones on the cell morphology and membrane integrity of *F. graminearum*. Collectively, this study aims to provide a theoretical basis for the targeted application of *Bacillus velezensis* DJ1 and its polyketone metabolites in the biological control of corn stalk rot.

## 2. Materials and Methods

### 2.1. Microbiological Material

The bacterial strain utilised in this study was the patented strain *Bacillus velezensis* DJ1 (CGMCC NO.25972), which was isolated from *Brassica rapa* var. *glabra* rhizosphere soil previously. This strain is typically cultivated in LB at 37 °C. The phytopathogenic fungus *F. graminearum* was maintained in our laboratory and cultivated on Potato Dextrose Agar (PDA) and Potato Dextrose Broth (PDB) at 28 °C. The corn seeds (*Zea mays* L. cv. Zhengdan 958) used in this experiment were purchased from a local agricultural market.

### 2.2. Polyketones Production

LB medium was utilized for polyketone production. One single colony was picked and inoculated into LB medium, followed by incubation at 37 °C for 12 h to obtain the seed culture. This seed culture was then inoculated into fresh LB medium at a 2% (*v*/*v*) inoculum rate for fermentation. After incubation at 37 °C with 180 rpm shaking for 48 h, the broth was subjected to centrifugation at 8000× *g* for 15 min at 4 °C. The supernatant was combined with isopropanol in a 1:4 (*v*/*v*) ratio and extracted for 2 h with constant stirring. The organic phase was concentrated by rotary evaporation under reduced pressure at 40 °C. The resultant precipitate was reconstituted in a minimal volume of methanol for subsequent analysis.

### 2.3. Antifungal Activity of the DJ1 Polyketones

Assays to evaluate antifungal activity were performed on PDA plates amended with different concentrations (0.625, 1.25, 2.5, 5, 10, 20, and 25 mg mL^−1^, *w*/*v*) of the polyketone extract. The concentration gradient was determined based on a preliminary experiment. We first tested a broad range of concentrations (0.125–50 mg mL^−1^), which revealed that concentrations below 0.625 mg mL^−1^ exerted no observable inhibitory effect on the mycelial growth of *F. graminearum*, while concentrations above 25 mg mL^−1^ did not further enhance the inhibitory effect. To fully characterize the dose-dependent relationship, we therefore selected 7 concentrations within the range of 0.625–25 mg mL^−1^. Control plates were added with the same volume of methanol as the extract. Each plate was centrally inoculated with a 7 mm mycelial plug of *F. graminearum* and incubated in darkness at 28 °C for 7 days. The inhibition rate of mycelial growth (in percentage) was calculated by applying the corresponding formula [23]:

Inhibition rate (%) = [(Dc − Dt)/Dc] × 100%, (1)

In the formula, Dc stands for the average mycelial diameter (mm) of control plates, and Dt refers to the average mycelial diameter on plates treated with the polyketone extract. Furthermore, the minimal inhibitory concentration (MIC) and minimum fungicidal concentration (MFC) were also quantified to evaluate the antifungal potency of the extract [24,25].

### 2.4. Inhibitory Mechanism of Polyketone of DJ1 on F. graminearum

#### 2.4.1. Effects of Polyketone of DJ1 on Mycelium Morphology of *F. graminearum*

Morphological changes in *F. graminearum* mycelium after treatment with crude polyketone extract of DJ1 were observed via SEM, referring to Wang et al. [26] with modifications. Briefly, filter-sterilized crude polyketones extract of DJ1 was added to PDA medium to a final concentration of MIC, PDA with equal methanol served as control. A 7 mm actively growing *F. graminearum* mycelial disc was inoculated on each plate, incubated at 28 °C. Inhibited mycelia and control mycelia were excised. Samples were fixed in 2.5% glutaraldehyde at 4 °C for 24 h. After dehydration through a graded ethanol series, the prepared samples were then sputter-coated with gold-palladium and examined using SEM (HITACHI, H-600, Hitachi High-Tech Co., Ltd., Tokyo, Japan) to assess morphological changes.

#### 2.4.2. Effects of Polyketones of DJ1 on Plasma Membrane Damage

To investigate the impact of polyketones of DJ1 on the membrane integrity of *F. graminearum*, nucleic acid and protein leakage were detected using the method described by Liu et al. [27]. In brief, *F. graminearum* spores (1 × 10^6^ CFU/mL) were inoculated into PDB containing the polyketones at 1 × MIC and 2 × MIC, and incubated at 28 °C for 48 h. The control group consisted of PDB cultures without polyketones addition. At the end of the incubation period, the cultures were subjected to centrifugation at 8000× *g* for 10 min. The collected supernatant was analyzed for OD_260_ and OD_280_ values to assess the extent of nucleic acid and protein leakage.

#### 2.4.3. Determination of Enzyme Activities of *F. graminearum*

Eight pathogen mycelial plugs (7 mm in diameter) were inoculated into 500 mL of PDB medium, followed by incubation at 28 °C with constant shaking at 180 rpm for 3 days. After mycelial growth was observed, 1 mL of the mycelial suspension was transferred to 100 mL of fresh PDB medium and further incubated under the same conditions for another 3 d. Subsequently, the extract was added to the culture to achieve final concentrations of 0 and 1 × MIC, respectively. The cultures were continuously shaken at 28 °C and sampled at 0, 12, 24, and 48 h post-extract addition. The activities of phenylalaninammo-nialyase (PAL), superoxide dismutase (SOD) and peroxidase (POD) were determined following the manufacturer’s instructions of the corresponding assay kits. Each treatment was performed with three biological replicates.

### 2.5. Greenhouse Experiment

The efficacy of polyketone of DJ1 against corn stalk rot induced by *F. graminearum* was evaluated in vivo. A pot experiment was conducted under greenhouse conditions with temperature at 25 °C, relative humidity ranging from 60 to 70%, and a Light—Dark ratio = 14:10 h [28]. All treatments were conducted using three-leaf stage corn plants, with 20 plants per treatment and three biological replicates. Three treatments were set up in the experiment as follows: a. maize plants were only inoculated with the spore suspension of the pathogen (1 × 10^6^ spores·mL^−1^); b. maize plants were sprayed with the polyketone extract, and 24 h later, inoculated with the pathogen spore suspension (1 × 10^6^ spores·mL^−1^); c. Sterile water inoculation (CK); d. 25% fludioxonil suspensions (fungicide control) [29].

All treated plants were incubated at 30 °C with high humidity [28]. The basal stem injection method was adopted for inoculation: a syringe was used to inject the pathogen spore suspension (or sterile water for the control group) into the basal stem tissue of maize. The injection wounds were covered with plastic wrap to maintain moisture, and the plants were observed regularly until disease symptoms appeared. Disease assessment was performed based on the grading standard for maize stalk rot [29]. The disease incidence, disease index and control efficacy were calculated.

Percentage of disease incidence (%) = (Total number of diseased seedlings/ Total number of emerged seedlings) × 100%(2)

Disease index = ∑[(Disease rating × Number of plants with the corresponding rating)/(Total number of assessed plants × Maximum disease rating)] × 100(3)

Control efficacy (%) = [(Disease incidence in the control group − Disease incidence in the treatment group)/Disease incidence in the control group] × 100%(4)

### 2.6. Soil Metagenomic Sequencing

To further explore the mechanism underlying the control of maize stalk rot by the polyketone extract of DJ1, soil metagenomic sequencing was conducted on the corn rhizosphere soil. Two treatments were established in the experiment: the polyketone-treated group (designated as “T”) and the equal-volume methanol-treated group (served as the control, designated as “CK”). Each treatment included 5 biological replicates with 20 plants. At 30 days post-inoculation (dpi), rhizosphere soil was collected, and soil DNA was extracted for high-throughput sequencing (Meige Biotechnology Co., Ltd., Guangzhou, China). Bacterial amplifications were performed using the universal primer pairs 341F/805R (Table A1) and the libraries were loaded into Illumina HiSeq. Analyses of the soil microbial community structure and functional profiles were conducted in accordance with the protocols described by Hu et al. [30].

### 2.7. Identification of DJ1 Polyketones

The extraction of target polyketones was carried out using a 10% (*v*/*v*) methanol solution. Subsequent analytical and purification procedures were performed using a high-performance liquid chromatography (HPLC) system (1220 Infinity II Analytical HPLC Purification System, Agilent, Shanghai, China) equipped with a reversed-phase C18 column (Agilent Prep 100 Å C18, 30 × 250 mm, 10 μm). The chromatographic conditions were as follows: the mobile phase consisted of CH_3_OH (containing 0.1% formic acid) and ddH_2_O (containing 0.1% formic acid); the flow rate was 1 mL/min; and the detection wavelength was 273 nm [22,31]. The detailed chromatographic separation program is provided in Table 1.

For structural identification, the HPLC system was coupled online with a mass spectrometry (MS) system (SYNAPT G2 HDMS Q-TOF, Waters, Milford, MA, USA). Mass spectrometric analysis was conducted in the negative electrospray ionization mode (ESI^−^). All acquired mass spectral data were processed using MassLynx™ software 4.1 (Waters, Milford, MA, USA).

### 2.8. Data Analysis

Statistical analysis of the experimental data was conducted employing SPSS 26.0 (IBM, Armonk, NY, USA), applying a significance threshold of *p* < 0.05. To assess significant differences between groups, Duncan’s multiple range test was implemented. Data visualization was accomplished using GraphPad Prism 8.0.

## 3. Results

### 3.1. Antifungal Activity Assay of the Polyketone Produced by DJ1

**Figure 1:**
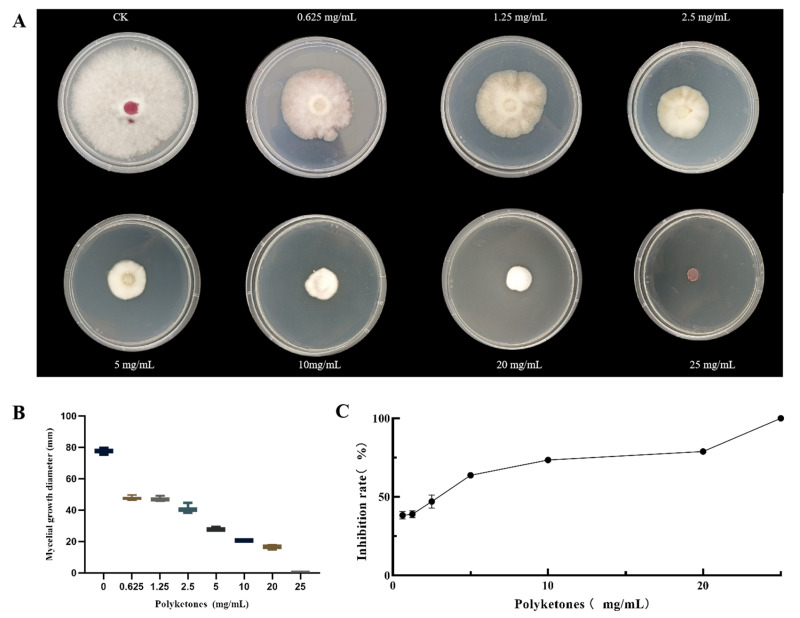
Antifungal Activity Assay of the polyketone Produced by DJ1. (**A**) Mycelial growth of *F. graminearum* on PDA medium supplemented with different concentrations of polyketones; (**B**) mycelial growth diameter of *F. graminearum* at different polyketones concentrations; (**C**) dose–response curve of mycelial growth inhibition rate of *F. graminearum* treated with various concentrations of polyketones; CK: control group (PDA without polyketones).

The effect of different concentrations of polyketone extract on the growth diameter of *F. graminearum* mycelium is dose-dependent. As shown in Figure 1A,B, the diameter of the mycelium decreased significantly as the concentration of the polyketone extract increased. On the 2nd day of incubation, the inhibition rates of the medium supplemented with the extract at concentrations of 25.0, 20.0, 10.0, and 5.0 mg/mL against *F. graminearum* all reached 100%. Accordingly, MIC was determined to be 5 mg/mL. After 7 days of incubation, the inhibition rate of the medium containing the extract at a concentration of 25.0 mg/mL against *F. graminearum* was 100%. Thus, the MFC was confirmed to be 25.0 mg/mL (Figure 1C).

### 3.2. Inhibitory Mechanism of Polyketone of DJ1 on F. graminearum

#### 3.2.1. Effect of DJ1 Polyketone on *F. graminearum* Mycelial Morphology

**Figure 2:**
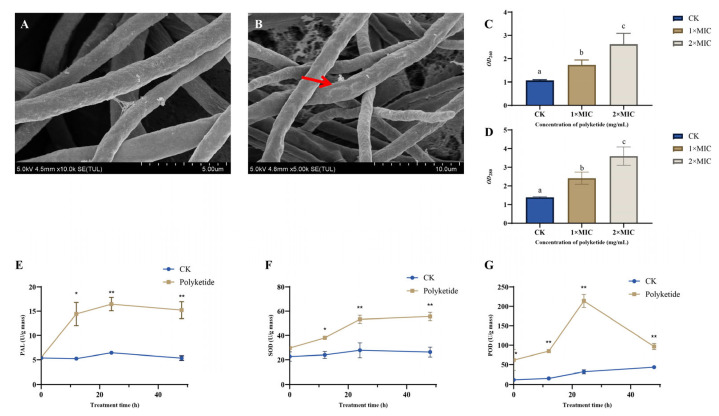
The antifungal mechanism of DJ1 polyketones against fungal hyphae. (**A**) SEM image of *F. graminearum* mycelia in CK; (**B**) SEM image of *F. graminearum* mycelia treated with polyketones, red arrow shows destruction of mycelial structure; (**C**) absorbance at 260 nm; (**D**) absorbance at 280 nm; (**E**) PAL activity; (**F**) SOD activity; (**G**) POD activity. CK: control group (without polyketones). Different lowercase letters indicate significant differences among groups at *p* < 0.05; * and ** indicate significant differences between T and CK groups at *p* < 0.05 and *p* < 0.01, respectively.

SEM images illustrating the morphological changes in *F. graminearum* following treatment with polyketones extracts from DJ1 are presented in Figure 2A,B. When *F. graminearum* mycelia were exposed to the minimum inhibitory concentration (MIC, 5 mg mL^−1^) of DJ1 polyketones, significant morphological alterations of the pathogen were observed. In the absence of polykeones treatment, *F. graminearum* hyphae exhibited normal growth with intact and healthy mycelial structures (Figure 2A). In contrast, after exposure to the DJ1 polyketone, the hyphae showed obvious collapse, and their cellular structures were disrupted (Figure 2B).

#### 3.2.2. DJ1 Polyketones Disrupts the Cell Membrane Integrity of *F. graminearum*

The results of biochemical analysis indicated that the amount of cell leakage increased significantly in a dose-dependent manner, and this conclusion could be confirmed by the increase in the levels of nucleic acids (*OD*_260_ nm) and proteins (*OD*_280_ nm) in the extracellular medium (Figure 2C,D).

The PAL activity in *F. graminearum* mycelia in the treated group was significantly higher than that in the CK group throughout the treatment period (Figure 2E). At each time point, the treated group showed a notable increase in PAL activity, suggesting that polyketones treatment induced the activation of the phenylalanine ammonia-lyase pathway in *F. graminearum* mycelia. The SOD activity in the treated *F. graminearum* mycelia was significantly elevated compared to the CK group (Figure 2F), indicating that polyketone treatment activated the antioxidant enzyme system of *F. graminearum*, yet the increased SOD activity might still be insufficient to counteract the oxidative damage caused by polyketones. The POD activity in *F. graminearum* mycelia treated with polyketones exhibited a significant and dynamic change compared to the CK group. POD activity in the treated group increased rapidly, reached a peak at around 24 h, and then decreased but still remained significantly higher than that in the CK group (Figure 2G). This indicates that polyketone treatment strongly affected the peroxidase system of *F. graminearum*, leading to abnormal fluctuations in POD activity.

### 3.3. DJ1 Polyketones Inhibit the Occurrence of Maize Stalk Rot in Greenhouse

The biocontrol efficacy of DJ1 polyketones against corn stalk rot at the seedling stage under greenhouse conditions was determined. As shown in Table 2, corn plants treated with DJ1 polyketones and fludioxonil had significantly lower disease incidence and disease index values than the *F. graminearum* group. Compared with the CK group, in the group inoculated with *F. graminearum*, the inner tissues of maize stems turned black, accompanied by severe rot at the inoculation site. In contrast, the group treated with the polykeones prior to *F. graminearum* inoculation showed a significant reduction in disease incidence, with a biocontrol efficacy of 44.33%. However, no significant difference was detected in biocontrol efficacy between the polyketones group and the fungicide treated group.

### 3.4. The Effect of DJ1 Polyketones on Bacterial Community Structure

**Figure 3:**
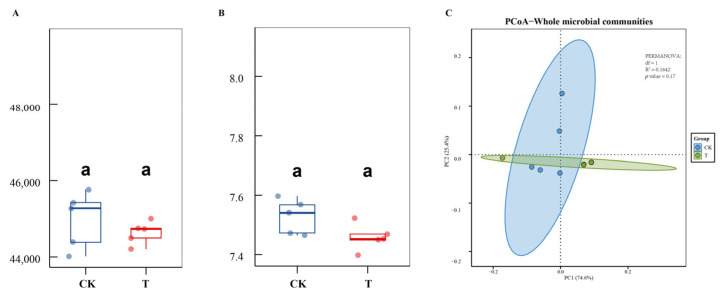
Influence of DJ1 polyketones on root-colonized microbiota. (**A**) Chao1 index; (**B**) Shannon index; (**C**) PCoA analysis are plotted based on Bray–Curtis distance metrices. The same letter ”a” indicated there are no significant difference in the Chao1 index and Shannon index between CK and T groups (*p* > 0.05).

A total of 443,923,398 bacterial 16S rRNA gene sequences were generated after filtering and quality control (Table A2). The α-diversity of the bacterial community was calculated based on species richness (Chao1 index) and comprehensive diversity (Shannon index) at the OTU level (Figure 3A,B). The Chao1 index reflects the total number of species in the community (richness), while the Shannon index integrates both species richness and evenness (the uniformity of individual distribution across species). Inoculation with DJ1 polyketones had no significant effects on the richness and microbial diversity of the microbial community (*p* > 0.05). For a better understanding of the impact of DJ1 polyketones inoculation on the microbial community, PCoA (Principal Coordinate Analysis) was used to examine β-diversity (Figure 3C). In PCoA, PC1 (the first principal coordinate) and PC2 (the second principal coordinate) represent the two dimensions that explain the largest proportions of variance in bacterial community composition. Specifically, PC1 accounted for 74.6% of the total variance, and PC2 accounted for 25.4%. There was no significant difference in the overall microbial community structure between the CK and T groups (*p* > 0.05).

**Figure 4:**
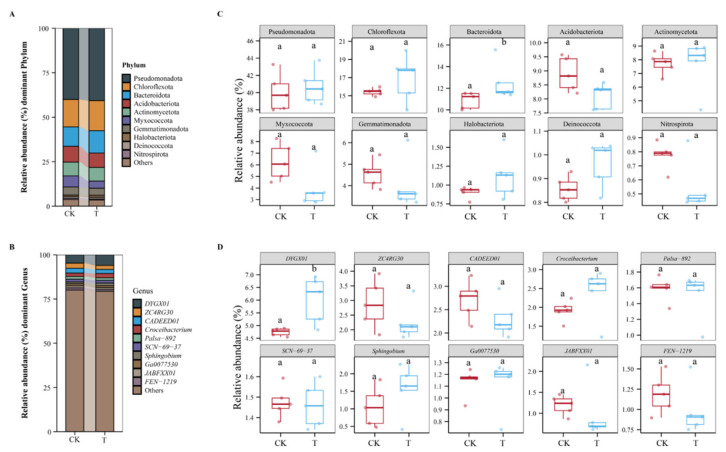
Evaluation of the species composition of the rhizosphere bacterial community across phylum and genus levels. (**A**) Relative abundance of bacterial phyla level; (**B**) relative abundance of bacterial genera level; (**C**) differences in the relative abundances of the top 10 bacterial groups among groups at the phylum level; (**D**) differences in the relative abundances of the top 10 bacterial groups among groups at the genus level. Different lowercase letters indicate significant differences between groups. CK indicates the control group and T indicates the group treated with polyketones.

The results of analyses of bacterial community abundance at the phylum and genus levels are shown in Figure 4. In both the CK and T groups, the phylum Pseudomonadota and the genus *DYGX01* exhibited the highest abundance (Figure 4A,B). Figure 4C displays the differences in the relative abundances of the top 10 bacterial phyla between the CK and T groups. For most phyla, there were no significant differences in relative abundance between 2 groups. However, for Bacteroidota, the relative abundance in the T group was significantly higher than that in the CK group. The differences in the relative abundances of the top 10 bacterial genera between the CK and T groups were shown in Figure 4D. Among these genera, only the relative abundance of *DYGX01* showed a significant difference between the two groups. The relative abundance of *DYGX01* in the T group was significantly higher than that in the CK group. For the other genera, there were no significant differences in relative abundance between the CK and T groups.

### 3.5. Identification of the Active Compounds

**Figure 5:**
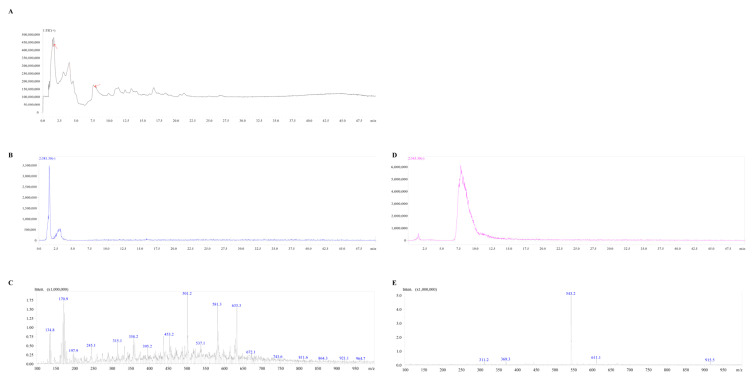
Isolation and identification of crude polyketones extract from DJ1. (**A**) HPLC chromatogram of crude polyketones extract from DJ1, red arrows represent two active substance peaks; (**B**) Re-purification chromatogram of bioactive component 1; (**C**) MS analysis result of bioactive component 1; (**D**) Re-purification chromatogram of bioactive component 2; (**E**) MS analysis result of bioactive component 2.

To isolate and identify the antifungal polyketone components from the crude polyketone extract of DJ1 with inhibitory activity against *F. graminearum*, an activity-guided fractionation strategy was employed. Through sequential fractionation and antifungal activity assays, the active fraction was successfully obtained. Subsequently, the active fraction was subjected to HPLC to evaluate its purity and separate potential components. The HPLC chromatogram revealed two distinct peaks corresponding to target components in the active fraction (Figure 5A). To further confirm purity, these two components were individually collected and re-analyzed by HPLC under the same chromatographic conditions. Both components displayed symmetric peak shapes with no detectable impurity peak, indicating that the two target components had been purified to a high degree (Figure 5B,D).

For structural identification of the purified components, MS detection was performed. By comparing the obtained mass spectral data with reference data from standard compounds and existing literature, the two purified components were ultimately identified as difficidin (MW 544) and bacillaene (MW 582), respectively (Figure 5C,E).

## 4. Discussion

Corn is highly susceptible to infections by *F. graminearum*, which not only causes severe maize stalk rot during the growing season but also leads to significant postharvest losses and substantial economic impacts on global corn production [32]. Against the backdrop of growing concerns over the evolution of chemical fungicide resistance in *F. graminearum* populations and the ecological risks associated with excessive fungicide use, microbial biocontrol strategies have emerged as sustainable alternatives for disease management. *Bacillus* spp. are well-documented for producing diverse antimicrobial metabolites [33,34], and this study focused on elucidating the role of polyketones from *B. velezensis* DJ1 (CGMCC NO.25972) in corn stalk rot biocontrol, with discussions centered on mechanistic significance, ecological safety, and practical agricultural applications.

The results showed that the growth inhibition of *F. graminearum* by DJ1-derived polyketones exhibited a significant dose-dependent effect (Figure 1), with MIC of 5 mg/mL and MFC of 25 mg/mL. This activity level is comparable to the inhibitory effects of Bacillus-derived antimicrobial metabolites against Fusarium pathogens reported in previous studies [35]. Notably, after 2 days of cultivation, the inhibition rates of *F. graminearum* by polyketones at concentrations of 25.0, 20.0, 10.0, and 5.0 mg/mL all reached 100%; however, after 7 days of cultivation, only the group treated with 25.0 mg/mL maintained a 100% inhibitory effect. This phenomenon indicates that high-concentration polyketones can completely block the reproductive cycle of the pathogen through a sustained effect, while the reduced inhibitory effect in low-concentration groups may be attributed to the degradation of polyketones or the adaptive stress response of the pathogen. These results provide direct experimental evidence for optimizing the application concentration in subsequent plants applications.

At the level of antifungal mechanism, SEM observations revealed that after treatment with polyketones at the MIC, the hyphae of *F. graminearum* exhibited obvious collapse, and the integrity of cell structure was destroyed (Figure 2A,B). This directly confirms that the polyketones exert an inhibitory effect by disrupting the cell morphology of the pathogen, which is consistent with our previous studies [36]. Further biochemical analysis showed that with the increase in polyketones concentration, the leakage of extracellular nucleic acids and proteins from *F. graminearum* increased significantly (Figure 2C,D), showing a dose-dependent characteristic. As the core barrier for cell material exchange and signal transmission, the impairment of cell membrane integrity leads to the massive loss of key intracellular biomacromolecules, ultimately causing metabolic disorders and even death of the pathogen [37]. This mechanism is consistent with the mode by which antibiotics produced by *Bacillus* spp. damages the cell membrane of fungal [38,39], suggesting that cell membrane damage may be one of the common mechanisms by which antimicrobial metabolites derived from *Bacillus* inhibit filamentous fungi.

In addition, the regulation of key enzyme activities in *F. graminearu* by DJ1-derived polyketones further reveals the complexity of their antifungal mechanism. Throughout the treatment period, *F. graminearum* in the treatment group exhibited significantly higher PAL activity compared to the CK (Figure 2E). PAL, a critical rate-limiting enzyme in the phenylpropanoid pathway, is intimately linked to both cell wall synthesis in pathogens and the biosynthesis of stress-responsive substances [40], including melanin, which reinforces cell wall rigidity to resist environmental stress and toxins. The abnormal increase in its activity may reflect the pathogen’s attempt to cope with polyketones stress by activating metabolic pathways, but this stress response ultimately failed to offset the inhibitory effect of the polyketones. The activity of SOD was significantly upregulated in the treatment group (Figure 2F), indicating that the pathogen activated the antioxidant defense system to scavenge reactive oxygen species (ROS) induced by polyketones; however, the increase in SOD activity may not be sufficient to completely scavenge excessive ROS, and the continuous accumulation of ROS still causes oxidative damage to the pathogen [41], exacerbating the membrane disruption already induced by polyketones. POD plays dual roles in pathogens: it collaborates with SOD to scavenge hydrogen peroxide (H_2_O_2_) and participates in cell wall lignification. The activity of POD showed a dynamic change of first increase and then decrease and was always higher than that in the control group (Figure 2G). The increase in POD activity in the early stage of stress may be an adaptive response of the pathogen to enhance antioxidant capacity and repair cell wall damage via lignification, while the decrease in activity in the later stage may be related to the loss of enzyme synthesis raw materials due to cell membrane damage or the destruction of enzyme structure by ROS. The abnormal changes in the above enzyme activities collectively constitute an important pathway for polyketones to inhibit pathogen growth at the metabolic interference level, and also explain why the polyketones can achieve efficient inhibition of *F. graminearum* from both the dual dimensions of cell morphology and physiological metabolism.

Greenhouse pot experiment results demonstrated that DJ1-derived polyketones effectively controlled corn stalk rot, with no significant difference in disease control efficacy compared to the chemical fungicide fludioxonil (Table 2). This finding directly indicates that DJ1 polyketones possess considerable potential as effective alternatives to chemical fungicides. Nevertheless, it is critical to acknowledge the limitations of greenhouse conditions, where environmental factors are strictly regulated and differ substantially from the uncontrollable, fluctuating conditions in open-field environments. Therefore, subsequent field-scale trials are essential to further validate the performance of DJ1 polyketones under real agricultural production conditions, which will provide more robust evidence for their practical application in sustainable corn stalk rot management.

The findings from this study explored the influence of DJ1 polyketones inoculation on the rhizosphere bacterial community. Firstly, regarding microbial diversity, both α-diversity and β-diversity analyses indicated that DJ1 polyketones inoculation did not exert a significant impact on the overall richness, diversity, or structure of the bacterial community (Figure 4). This implies that DJ1 polyketones are relatively benign to the native microbial community, which is a favorable characteristic as it reduces the risk of disrupting the inherent ecological balance of the soil. When focusing on the taxonomic composition at the phylum and genus levels, although the dominant phylum Pseudomonadota and genus *DYGX01* remained prevalent in both the control (CK) and treatment (T) groups, notable changes were observed in specific taxa. The significant increase in the relative abundance of Bacteroidota in the T group is noteworthy. Bacteroidota members are often involved in organic matter degradation, particularly in the breakdown of complex carbohydrates [42]. This elevation might suggest that DJ1 polyketones could potentially enhance the soil’s capacity for decomposing certain organic substances, which could have positive implications for nutrient cycling. Additionally, the marked rise in *DYGX01* abundance in the T group points to a specific response of this genus to DJ1 polyketones. Notably, to our knowledge, there are currently no direct reports on the functional traits or ecological roles of *DYGX01* in existing literature, which represents a key research gap in the study of rhizosphere microbiota associated with corn stalk rot biocontrol. Further investigation into the functional traits of *DYGX01* could unveil its role in the rhizosphere, such as in plant-microbe interactions or nutrient acquisition, and how the DJ1 polyketone modulates this role. In summary, while DJ1 polyketones do not drastically alter the overall microbial community, they do induce targeted changes in specific bacterial taxa. These targeted changes could have cascading effects on soil processes and plant health, warranting further exploration into the functional consequences of these taxonomic shifts.

*Bacillus* spp. are renowned for their ability to produce diverse secondary metabolites [19,43,44]. In this study, two active components from DJ1’s crude extract were identified as difficidin (MW 544) and bacillaene (MW 582) via MS analysis (Figure 5), consistent with our previous whole-genome sequencing results that predicted polyketone synthase gene clusters in DJ1. This finding aligns with related studies [18,19,45], highlighting the conserved role of difficidin and bacillaene in antifungal defense among *Bacillus* strains. Although difficidin and bacillaene have been well-documented for their potent antifungal activity against plant pathogens in previous research, their poor stability under field conditions has become a critical bottleneck restricting their large-scale agricultural application. To tackle this problem, it is necessary to develop targeted technical strategies. First, optimize formulation technologies to establish a protective barrier for the two active components. Building on existing formulation explorations in this study, more biocompatible and degradation-resistant carriers can be selected, such as modified chitosan [46] or sodium alginate-montmorillonite [47] composite carriers, to replace traditional inert carriers like kaolin. These modified formulations not only reduce thermal/photodegradation-induced activity loss of difficidin and bacillaene but also enable sustained release in soil, while lowering the frequency of application. Second, conduct targeted molecular structure modification of difficidin and bacillaene. Guided by their structure-activity relationships, modifying non-essential functional groups with stable moieties can enhance the components’ resistance to soil enzymatic degradation without compromising their antifungal activity. These stability-focused strategies address the core limitation of difficidin and bacillaene while preserving the ecological safety of DJ1 polyketones, thereby providing reliable support for the sustainable management of corn stalk rot.

## 5. Conclusions

This research systematically explored the antifungal potential and mechanism of polyketones derived from *B. velezensis* DJ1 against *F. graminearum*, the causal agent of corn stalk rot. Results demonstrated that DJ1 polyketones exhibited dose-dependent antifungal activity, with MIC of 5 mg/mL and MFC of 25 mg/mL. These polyketones disrupted *F. graminearum* mycelial morphology, impaired cell membrane integrity, by increasing nucleic acid and protein leakage, and caused oxidative damage in the fungus. In greenhouse experiments, DJ1 polyketones reduced maize stalk rot incidence with a control efficacy of 44.33%. Soil metagenomic analysis revealed that DJ1 polyketones did not significantly disturb the overall diversity or structure of the rhizosphere bacterial community but specifically increased the relative abundance of Bacteroidota and the genus *DYGX01*. HPLC-HDMS Q-TOF identification confirmed the active components as difficidin and bacillaene. Collectively, these findings validate that DJ1-derived polyketones are promising biocontrol agents for corn stalk rot, offering an environmentally friendly alternative to chemical fungicides and providing a theoretical basis for their application in sustainable agricultural disease management.

## Figures and Tables

**Table 1:** HPLC separation conditions in this study.

Time (min)	Methanol Concentration (%)
0–5	20
5–50	20–90 (linear gradient)
50–55	100

**Table 2:** Effect of DJ1 polyketone against corn stalk rot in greenhouse.

Treatments	Disease Incidence (%)	Diease Index	Disease Control Efficacy (%)
DJ1 polyketone	44.89 ± 6.28 b	23.68 ± 2.32 b	44.33 ± 4.82 a
Fludioxonil	50.62 ± 5.44 b	24.65 ± 1.43 b	37.22 ± 5.02 a
*F. graminearum*	80.63 ± 2.73 a	80.26 ± 2.82 a	--

Note: Values are the means ± standard error of 3 replicates. Different lowercase letters in the same column indicate significant differences among treatments at the *p* < 0.05 level.

## Data Availability

The supporting data used in this study are available from the corresponding author upon request.

## Appendix A

**Table A1:** Primer sequences used for microbial community analysis.

Microbes	Target Gene	Primer Sequences (5′to 3′)
Bacteria	16S rRNA	341F-CCTACGGGNGGCWGCAG 805R-GACTACHVGGGTATCTAATCC

**Table A2:** Read number of the amplicon analysis in this study.

Sample	Total Reads	Total Bases	Total Reads with Ns	N Reads%	N%	Error%	Q20%	Q30%	GC%
CK-1	46,001,828	6,841,198,840	72,051	0.16	0	0.0239	98.21	94.79	61.07
CK-2	45,867,046	6,820,133,424	75,292	0.16	0	0.024	98.19	94.74	61.05
CK-3	42,927,794	6,383,065,139	61,364	0.14	0	0.024	98.18	94.7	61.41
CK-4	49,209,824	7,316,867,623	70,382	0.14	0	0.024	98.15	94.64	61.52
CK-5	45,845,230	6,817,708,740	65,531	0.14	0	0.0238	98.21	94.8	61.33
T-1	35,992,282	5,349,384,452	53,976	0.15	0	0.0242	98.12	94.53	60.59
T-2	49,828,222	7,412,718,835	70,156	0.14	0	0.0236	98.25	94.91	61.27
T-3	40,778,120	6,068,850,552	53,300	0.13	0	0.0234	98.3	95.05	61.13
T-4	44,553,116	6,635,128,835	53,605	0.12	0	0.0228	98.45	95.49	61.03
T-5	42,919,936	6,385,262,071	59,633	0.14	0	0.0235	98.29	95.01	60.77
